# Supplementary material for: Low agreement and frequent invalid controls in two SARS-CoV-2 T-cell assays in people with compromised immune function
Source: PLoS One. 2025 Jan 24;20(1):e0317965. doi: 10.1371/journal.pone.0317965 (PMC11761106; doi:10.1371/journal.pone.0317965)

**Supplemental Data**

**Assessing the agreement of two SARS-CoV-2 T-cell assays in people with compromised immune function**

Annette Audigé, Alain Amstutz, Macé M. Schuurmans, Patrizia Amico, Dominique L. Braun, Marcel P. Stoeckle, Barbara Hasse,^,^ René Hage, Dominik Damm, Michael Tamm, Nicolas J. Mueller, Huldrych F. Günthard, Michael T. Koller, Christof M. Schönenberger, Alexandra Griessbach, Niklaus D. Labhardt, Roger D. Kouyos, Alexandra Trkola, Michael Huber, Katharina Kusejko, Heiner C. Bucher, Irene A. Abela, Matthias Briel, Frédérique Chammartin, Benjamin Speich, and the Swiss HIV Cohort Study, and the Swiss Transplant Cohort Study

**Supplement 1:**

**Eligibility Criteria**

Inclusion criteria:

- - Patients with either a HIV infection or recipients of solid organs registered in the SHCS and STCS cohorts and signed informed consent form
  - Patients aged ≥18 years
  - Patients receiving a new bivalent (Wuhan/Omicron BA.1) mRNA SARS-CoV-2 vaccine in the frame of clinical routine, according to the treating physician
  - Consent provided for the COVERALL observational bivalent booster extension study (third sub-protocol and usage of biological samples)

Exclusion criteria:

- Acute symptomatic SARS-CoV-2 infection, influenza or other acute respiratory tract infection
- Any emergency condition requiring immediate hospitalization for any condition
- Known allergy or contra-indications for vaccines or any vaccine components or any other contraindication to receive the vaccine according to the treating physician
- Patients who did not receive the “basic immunization” SARS-CoV-2 vaccination (e.g. two doses of Spikevax from Moderna, two doses of Comirnaty from Pfizer-BioNtech).

**Table S1: Baseline characteristics of the 81 study participants before receiving a bivalent mRNA SARS-CoV-2 vaccine**

| **Characteristics** | **People living with HIV (n=35)** | **Solid organ transplant recipients (n=46)** | **Total (n=81)** |
| --- | --- | --- | --- |
| **Median age (IQR)** | 58 (47-63) | 57 (47-64) | 57 (46-64) |
| **Sex** |  |  |  |
| Male | 30/35 (85.7%) | 28/46 (60.9%) | 58/81 (71.6%) |
| Female | 5/35 (14.3%) | 18/46 (39.1%) | 23/81 (28.4%) |
| **Vaccine** |  |  |  |
| mRNA-1273.214 by Moderna | 17/35 (48.6%) | 37/46 (80.4%) | 54/81 (66.7%) |
| BA.1–adapted BNT162b2 by Pfizer-BioNTech | 18/35 (51.4%) | 9/46 (19.6%) | 27/81 (33.3%) |
| **Antibody test to the nucleocapsid protein** |  |  |  |
| Non-reactive | 10/35 (28.6%) | 27/46 (58.7%) | 37/81 (45.7%) |
| Reactive | 25/35 (71.4%) | 18/46 (39.1%) | 43/81 (53.1%) |
| Missing | 0/35 (0.0%) | 1/46 (2.2%) | 1/81 (1.2%) |
| **History of cardiovascular disease or metabolic syndrome^a^** | 14/35 (40.0%) | 40/46 (97.0%) | 54/81 (66.7%) |
| **Number of previously received SARS-CoV-2 vaccines** |  |  |  |
| 2 | 1/35 (2.9%) | 2/46 (4.3%) | 3/81 (3.7%) |
| 3 | 32/35 (91.4%) | 31/46 (67.4%) | 63/81 (77.8%) |
| 4 | 2/35 (5.7%) | 12/46 (26.1%) | 14/81 (17.3%) |
| 5 | 0/35 (0.0%) | 1/46 (2.2%) | 1/81 (1.2%) |
| **Median days since last vaccination before receiving the bivalent vaccine (IQR)** | 323 (323-337) | 351 (286-383) | 333 (312-365) |
| **SARS-CoV-2 specific monoclonal antibodies received within the last 6 months^c^** | 0/35 (0.0%) | 4/46 (8.7%) | 4/81 (4.9%) |
| **CD4 cell count (cells/µL)^a^** |  |  |  |
| <350 | 11/35 (31.4%) | - |  |
| ≥350 | 24/35 (68.6%) | - |  |
| **Suppressed HIV viral load^ab^** | 35/35 (100.0%) | - |  |
| **Transplanted organ^c^** |  |  |  |
| Kidney transplant | - | 11/46 (23.9%) |  |
| Lung transplant | - | 35/46 (76.1%) |  |
| **Immunosuppressive therapy^c^** |  |  |  |
| Less intense (≤2 regimen)^d^ | - | 6/46 (13.0%) |  |
| Intense (3 or 4 regimen)^d^ | - | 40/46 (87.0%) |  |
| **Median days since transplant (IQR) ^c^** | - | 1219 (616-3008) |  |

^a^Only considering participants from the Swiss HIV Cohort Study

^b^Suppressed HIV viral load defined as <50 copies/ml

^c^Only considering participants from the Swiss Transplant Cohort Study

^d^Intense treatment defined as triple or quadruple immunosuppressive regimen vs. less intense immunosuppressive therapy defined as dual immunosuppressive regimen

Abbreviations: IQR=Interquartile range; SHCS=Swiss HIV Cohort Study; STCT= Swiss Transplant Cohort Study

**Table S2:** **Agreement between the SARS-CoV-2 IGRA by Euroimmun and the IGRA SARS‑CoV‑2 by Roche classifying Euroimmun borderline results as a positive response**

|  | **IGRA SARS-CoV-2 by Roche** | | | | |
| --- | --- | --- | --- | --- | --- |
| **SARS-CoV-2 IGRA by Euroimmun** |  | Positive | Negative | Invalid | **Total** |
|  | Positive | 105 | 1 | 11 | 117 (67.6%) |
|  | Negative | 12 | 6 | 0 | 18 (10.4%) |
|  | Invalid | 22 | 13 | 3 | 38 (22.0%) |
|  | **Total** | 139 (80.3%) | 20 (11.6%) | 14 (8.1%) | 173 (100.0%) |

κ = 0.20; overall percent agreement: 66%; expected agreement 57%.

Borderline results from the Euroimmun test were classified as a positive T-cell response.

**Table S3: Agreement between the SARS-CoV-2 IGRA by Euroimmun and the IGRA SARS‑CoV‑2 by Roche classifying borderline results as a negative response**

|  | **IGRA SARS-CoV-2 by Roche** | | | | |
| --- | --- | --- | --- | --- | --- |
| **SARS-CoV-2 IGRA by Euroimmun** |  | Positive | Negative | Invalid | **Total** |
|  | Positive | 93 | 1 | 11 | 105 (60.7%) |
|  | Negative | 24 | 6 | 0 | 30 (17.3%) |
|  | Invalid | 22 | 13 | 3 | 38 (22.0%) |
|  | **Total** | 139 (80.3%) | 20 (11.6%) | 14 (8.1%) | 173 (100.0%) |

κ = 0.14; overall percent agreement: 59%; expected agreement 53%.

Borderline results from the Euroimmun test were classified as a negative T-cell response.

**Table S4**: **Agreement between the SARS-CoV-2 IGRA by Euroimmun and the IGRA SARS‑CoV‑2 by Roche for each assessed time point**

| **Blood samples collected at baseline** | | | | | |
| --- | --- | --- | --- | --- | --- |
|  | **IGRA SARS-CoV-2 by Roche** | | | | |
| **SARS-CoV-2 IGRA by Euroimmun** |  | Positive | Negative | Invalid | **Total** |
|  | Positive | 20 | 0 | 1 | 21 (52.5%) |
|  | Negative | 4 | 2 | 0 | 6 (15.0%) |
|  | Invalid | 6 | 9 | 0 | 13 (32.5%) |
|  | **Total** | 30 (75.0%) | 9 (22.5%) | 1 (2.5%) | 40 (100.0%) |
| κ = 0.20; overall percent agreement : 55%; expected agreement: 44% | | | | | |
| **Blood samples collected at 4 weeks follow-up (i.e. 4 weeks after receiving bivalent SARS-CoV-2 vaccine)** | | | | | |
|  | **IGRA SARS-CoV-2 by Roche** | | | | |
| **SARS-CoV-2 IGRA by Euroimmun** |  | Positive | Negative | Invalid | **Total** |
|  | Positive | 44 | 1 | 4 | 49 (66.2%) |
|  | Negative | 7 | 2 | 0 | 9 (12.2%) |
|  | Invalid | 11 | 3 | 2 | 16 (21.6%) |
|  | **Total** | 62 (83.8%) | 6 (8.1%) | 6 (8.1%) | 74 (100.0%) |
| κ = 0.16; overall percent agreement : 65%; expected agreement: 58% | | | | | |
| **Blood samples collected at 6 months follow-up (i.e. 6 months after receiving bivalent SARS-CoV-2 vaccine)** | | | | | |
|  | **IGRA SARS-CoV-2 by Roche** | | | | |
| **SARS-CoV-2 IGRA by Euroimmun** |  | Positive | Negative | Invalid | **Total** |
|  | Positive | 41 | 0 | 6 | 47 (79.7%) |
|  | Negative | 1 | 2 | 0 | 3 (5.1%) |
|  | Invalid | 5 | 3 | 1 | 9 (15.3%) |
|  | **Total** | 47 (79.7%) | 5 (8.5%) | 7 (11.9%) | 59 (100.0%) |
| κ = 0.26; overall percent agreement: 75%; expected agreement: 56% | | | | | |

For calculating κ and agreements, borderline results from the Euroimmun test were classified as a positive T-cell response.

**Table S5: Baseline characteristics of the 81 study participants stratified by the number of invalid Roche and Euroimmun T-cell test results**

^a^Only considering participants from the Swiss HIV Cohort Study

^b^Suppressed HIV viral load defined as <50 copies/ml

^c^Only considering participants from the Swiss Transplant Cohort Study

^d^Intense treatment defined as triple or quadruple immunosuppressive regimen vs. less intense immunosuppressive therapy defined as dual immunosuppressive regimen

Abbreviations: IQR=Interquartile range; SHCS=Swiss HIV Cohort Study; STCT= Swiss Transplant Cohort Study

**Table S6: In depth analyses of invalid SARS-CoV-2 T-test results**

|  | **Invalid Euroimmun T-cell test**  **(n=38)** | | **Invalid Roche T-cell test**  **(n=14)** | |
| --- | --- | --- | --- | --- |
|  | **PWH**  **(n=2)** | **SOT recipients**  **(n=36)** | **PWH**  **(n=8)** | **SOT recipients**  **(n=6)** |
| **Invalid positive control** | 1/38 (2.6%) | 36/38 (94.7%) | 0/14 (0%) | 3/14 (21.4%) |
| Positive result for specific stimulation among samples with invalid positive control* | 1/1 (100%) | 13/36 (36.1%) | 0/0 (0%) | 1/3 (33.3%) |
| **Invalid negative control** | 1/38 (2.6%) | 0/38 (0%) | 8/14 (57.1%) | 3/14 (21.4%) |
| Positive result for specific stimulation among samples with invalid negative control* | 1/1 (100%) | 0/0 (0%) | 8/8 (100%) | 3/3 (100%) |

*Including borderline results for the Euroimmun T-cell test

**Figure S1: Interferon-γ concentrations of the positive and negative controls at different time points using either the Euroimmun or the Roche T-cell test**

**
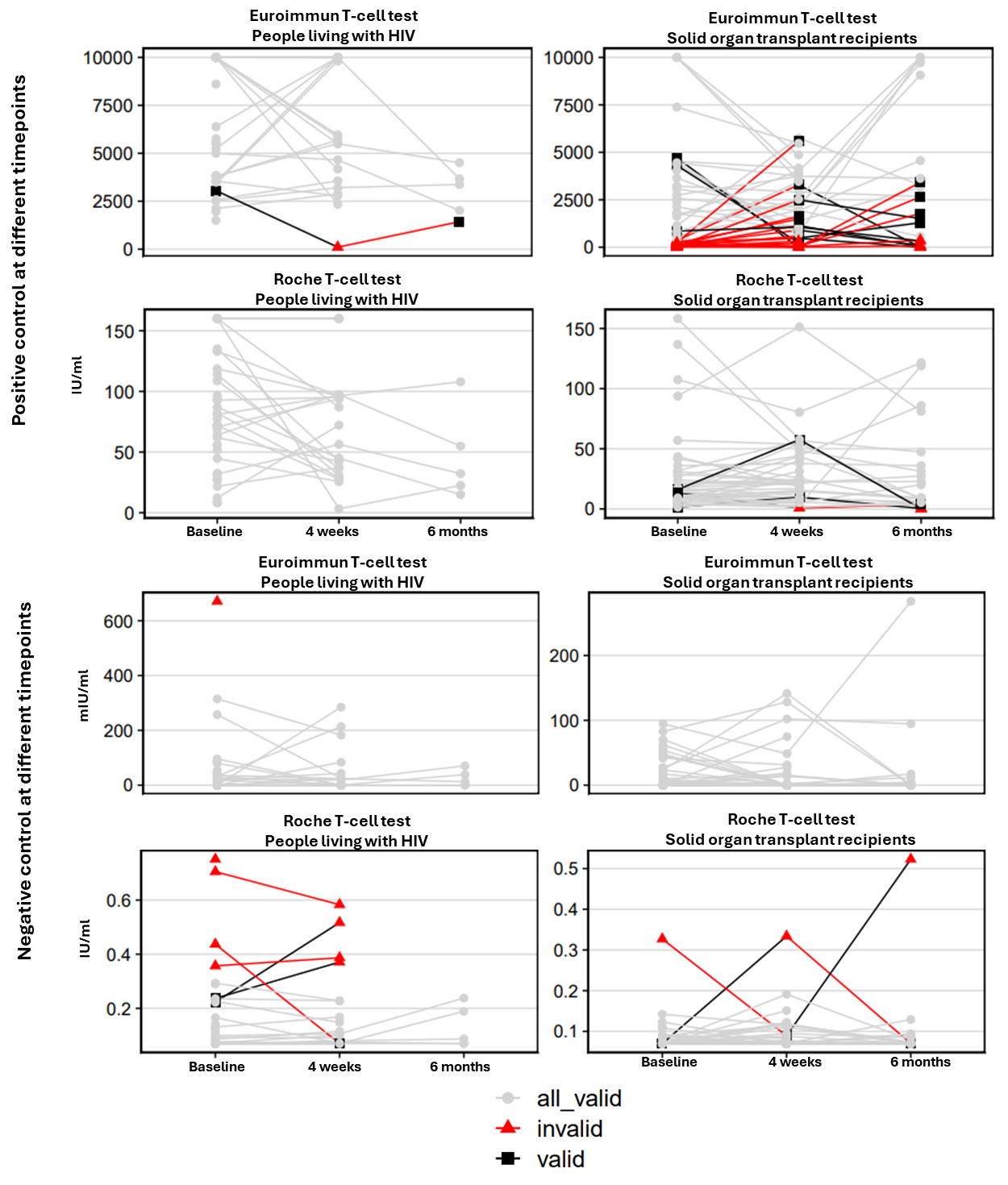
**

**Figure S2: Correlation of the positive control between the Euroimmun and the Roche T-cell test**


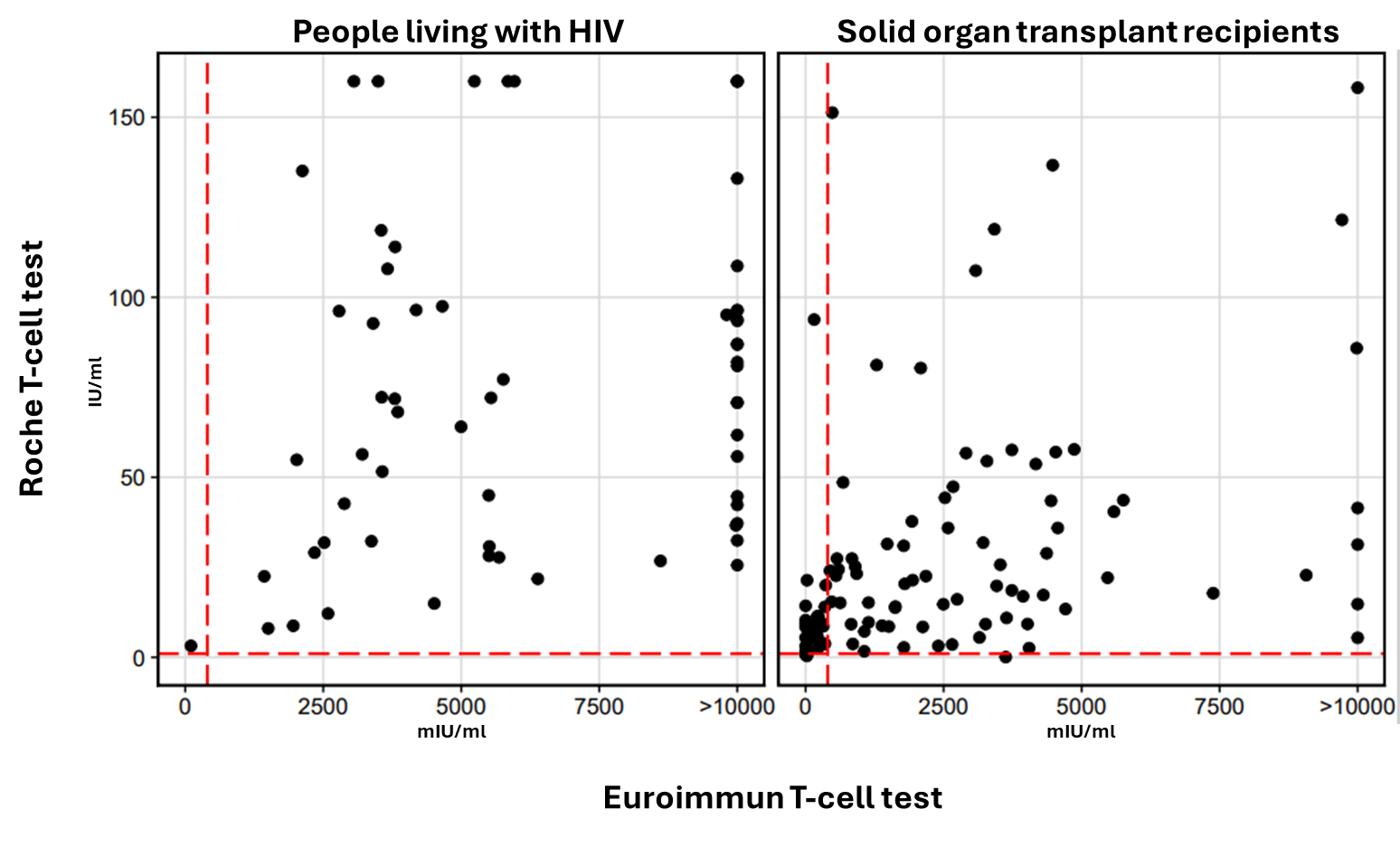

Supplement: S1 File — (DOCX) [file pone.0317965.s002.docx]
