# Supplementary material for: Low agreement and frequent invalid controls in two SARS-CoV-2 T-cell assays in people with compromised immune function
Source: PLoS One. 2025 Jan 24;20(1):e0317965. doi: 10.1371/journal.pone.0317965 (PMC11761106; doi:10.1371/journal.pone.0317965)
Supplement: Attachment — Submitted filename: PolsOne2024.docx [file pone.0317965.s003.docx]

Audigé et al. propose a study of T cell response to SARS-CoV2 in vaccinated immunocompromised patients: people living with HIV and solid organ transplant recipients.

The study is well presented and clear. However, several minor points needs to be clarified before publication:

- The threshold for positive response to the two tests need to be indicated
- The indeterminate results (or ‘invalid’) needs to be explained: is this a negative response to the positive control or a positive response to the negative control?
- The high proportion of negative results with the Euroimmun tests may be linked to the lack of SARS-Cov2 antigen stimulation (spike alone)

In the abstract, ‘vaccinated patients’ should be quoted. Also, ‘immunocompromised’ should replace ‘vulnerable’ patients.
